# China’s Carbon Emissions and Trading Pilot, Political Connection, and Innovation Input of Publicly Listed Private Firms

**DOI:** 10.3390/ijerph17176084

**Published:** 2020-08-21

**Authors:** Wei Liu, Chunquan Yu, Shixiong Cheng, Jingyi Xu, Yuzhao Wu

**Affiliations:** 1 Economics and Management School, Wuhan University, Wuhan 430072, China; liuweiabc9055@whu.edu.cn (W.L.); 2018201050045@whu.edu.cn (C.Y.); 2017201050167@whu.edu.cn (J.X.); 2019201050127@whu.edu.cn (Y.W.); 2 School of Business, Hubei University, Wuhan 430062, China; 3 Institute for Open Economy Research Centre, Hubei University, Wuhan 430062, China

**Keywords:** CCETP, publicly listed private firms, innovation input, political connection

## Abstract

Taking China’s carbon emissions and trading pilot (CCETP) as a quasi-natural experiment, this paper examines the impact of CCETP on publicly listed private firms’ innovation input and the moderating effect of the firms’ political connection based on the difference-in-differences model. The results show that CCETP has a significantly positive effect on the innovation input of Chinese publicly listed private firms. Moreover, the political connection of executives exhibits a positive moderating effect on CCETP’s impact on innovation input. Meanwhile, the effect is more significant in regions with high environmental protection investment and large publicly listed private firms. The conclusions could provide some policy enlightenment for China’s carbon market, as well as a rational adjustment of the relationship between political connection and innovation input of publicly listed private firms in the future.

## 1. Introduction

Over the past 40 years of reform and opening-up, China has gradually completed the transition from a planned economy to a market economy, which has an increasing impact on firm behavior. As one of the important components of the Chinese economy, private firms have experienced a leapfrog development process of growing from weak to strong, which plays an increasingly critical role in the improvement of Chinese firms’ innovation. According to the latest China Statistical Yearbook on Science and Technology (2018), the proportion of patent applications of private firms has reached 77.8% in China, which is 12 times that of state-owned firms (6.5%). Private firms have become the main force of innovation in China. Nevertheless, state-owned firms hold the lifeline of the national economy, and they occupy more national resources and capital than private ones in China. For the acquisition of important resources such as land and capital, state-owned firms often obtain them at a lower cost. Furthermore, state-owned firms have political advantages that private firms do not. They often have a monopoly advantage on important resources and key fields with the support of the government; therefore, they have huge advantages of scale. In contrast, private firms are at a relatively disadvantaged position in the market economy. Therefore, private firms have less ability to bear losses due to policy changes. It means that in the development of the government-dominated economy in China, private firms are more concerned about the impact of changes in government policies, such as minor changes in environmental regulation. Therefore, it is necessary to focus on the impact of environmental regulation on Chinese publicly listed private firms.

Among the existing literature, the impact of environmental regulation on firm innovation has always been one of the focuses. The weak Porter hypothesis argues that reasonable environmental regulation will guide firms to improve production strategies and stimulate firm innovation (Porter and Vanderlinde, 1995) [1]. Nevertheless, there is controversy in academia about the weak Porter hypothesis. Although extensive research on the European Union Emissions Trading Scheme (EU ETS) initially found that environmental regulation promoted firm innovation (Calel and Dechezlepretre, 2016) [2], more research believes that environmental regulation has a crowding-out effect on firm investment and inhibits firm innovation (Kneller and Manderson, 2012; Ramanathan et al., 2010) [3,4]. Subsequent research suggests that environmental regulation may have a nonlinear effect on firm innovation, which is presented as U-shaped (Ouyang et al., 2020; Pan et al., 2019) [5,6] or inverted U-shape (Wang and Shen, 2016) [7]. Especially under the influence of different mechanisms of administrative environmental regulation and market-based environmental regulation, different types of environmental regulation tools will have diverse impacts on firm innovation (Ambec et al., 2013; Jung et al., 1996; Stranlund, 1997) [8,9,10].

China’s government began to learn from the European Union and introduced a cap and trade system to reduce pollution in 2010, which is a market-based environmental regulation. In 2011, the National Development and Reform Commission in China released the Notice on Carrying Out the Pilot Project on Carbon Emissions Trading, approving seven carbon emissions and trading pilots to be established, which includes Beijing, Tianjin, Shanghai, Chongqing, Hubei, Guangdong, and Shenzhen. These seven pilots officially launched the carbon emissions and trading market from 2013 to 2014. The carbon market has begun to become an important factor affecting the revenue, cost, and various economic activities of the regulated firms included in the carbon emission quota’s management list of China. In 2017, China further proposed to start a national carbon market. Therefore, did CCETP have a promoting effect on China’s firm innovation, as expected by the weak Porter hypothesis? This question requires further empirical tests. Some scholars have tried to study the impact of the carbon market on firm innovation using invention patents, green patents, and so on (Feng et al., 2017; Zhang et al., 2019) [11,12]. However, their conclusions are quite different. This inconsistency may be explained by the fact that patents mainly measure firm innovation output; however, it cannot truly measure the direct impact of the carbon market on firm innovation due to the relative low innovation capacity and R&D efficiency of China’s firms, which lead to great uncertainty in the transition of innovation input into innovation output. Liu et al. (2020) [13] found that the R&D expenditure of industrial firms in China’s 31 provinces, autonomous regions, and municipalities directly under the central government has increased year by year, but the growth rate of R&D output was much lower than the growth rate of R&D investment. Chiu et al. (2012) [14] found that the R&D efficiency of the electronics and computer industries in China’s high-tech industries was low, mainly because some patents cannot effectively create value. Zhang et al. (2019) [15] found that the output efficiency of new products in 31 provinces, autonomous regions, and municipalities directly under the central government in Chinese mainland was not ideal. On the contrary, R&D investment can at least reflect the direct response of firms in innovation to the changes in environmental regulation. Accordingly, this paper emphasizes that, in China, variables that measure the innovation input of firms can more accurately reflect the direct effects of CCETP on firm innovation. Nevertheless, whether CCETP will exhibit distinctive effects on the innovation input (R&D investment) and the innovation output (patents) of firms should be further examined.

Existing literature mainly employs a full sample that includes all kinds of firms in China to analyze the impact of environmental regulation on firm innovation (Ouyang et al., 2020; Feng et al., 2017; Zhang et al., 2019) [5,11,12]. Most of them ignore the particularity of private firms. The difference in obtaining critical market resources and participating in market competition may affect firm innovation, especially during the transition of China from a planned economy to a market economy. For example, state-owned firms in China have innate ties with the government. Their executives generally have close contacts with the government, and they are more resistant to the risks of policy changes. In contrast, private firms without political connection are often in a weak position when acquiring critical resources and participating in market competition. However, the political connection of executives can help private firms establish the acquired associations with the government, which makes political associations have a more significant impact of environmental regulation on a firm’s innovation. We are thus motivated to focus on the impact of CCETP on the innovation of publicly listed private firms in China.

Moreover, existing studies have been conducted on the measurement and impact of political connection on the outcomes of environmental regulation. Zhan and Tang (2016) [16] divided the political connection into political relations, organizational relations, and personal relations. Among them, political connection means some of the firm’s executives are or have served in government departments or legislatures. However, more studies have measured the level of political connection using indicators such as the proportion of general directors with political backgrounds and whether chairmen or general managers have political experience (Fan et al., 2007; Liu et al., 2017) [17,18]. Some studies have also shown that political connection probably have different impacts on the effect of environmental regulation. On the one hand, a political connection may have a refuge effect on firms’ violation of environmental regulation because of the collusion between the government and the firms, which reduces the actual effect of environmental regulation (De Villiers et al., 2011) [19]. On the other hand, firms with political connection will perform environmental protection responsibilities as requirements of the government to attain favorable regulatory conditions (Agrawal and Knoeber, 2001) [20], alleviate financing constraints (Claessens et al., 2008; Li et al., 2008) [21,22], get government assistance (Faccio and Masulis, 2006) [23], and avoid negative impacts on firm stock returns and firm performance (Chang and Wong, 2004) [24], which may lead to environmental regulation playing an effective role. Then, will the differences in political connection have a moderating effect on the impact of CCETP on the innovation of publicly listed private firms? This question needs to be tested.

Our research’s main importance is to find the effect of market-based environmental regulation, which is CCETP, on the firms’ R&D investment and the moderating impact of the firms’ political connection. There are two contributions in this paper. First, this paper enriches the studies that discuss the short-term policy effect of CCETP from the perspective of publicly listed private firms’ R&D investment. Second, existing literature rarely explores the role of the political connection of the firms’ executives on the impact of CCETP on economic activities. This paper makes further examination of whether the differences in political connection have moderating effects on the impact of CCETP on publicly listed private firms’ innovation input or not.

The remainder of this paper is as follows. Section 2 provides the background of CCETP, mechanism analysis of its influence on firm innovation, and research hypotheses. Section 3 describes the methodology, variables, and model. Section 4 presents the baseline results and robustness checks. Section 5 outlines further analysis. Section 6 presents the conclusions and policy implications.

## 2. Background of CCETP Policy and Theoretical Mechanism

### 2.1. Background of CCETP Policy

In 2011, the Chinese government approved Beijing, Tianjin, Shanghai, Chongqing, Hubei, Guangdong, and Shenzhen as carbon emissions and trading pilots. Since then, the transaction amount in these carbon markets has steadily increased. By the end of 2018, the cumulative transaction volume of China’s carbon emission rights reached 270 million tons, and the cumulative transaction amount exceeded 6 billion yuan [25]. The basis of CCETP is the allocation of quotas, and the allocation methods of quotas in each pilot include two ways, which are free and paid. In order to determine the free carbon emission quotas, each pilot in China generally adopts the historical intensity method, that is, the average CO_2_ emission of firms in previous years is used as a benchmark value to determine free emission quotas. Specifically, first, the regulated firms report their historical data of carbon emission. Then, the Development and Reform Commissions of each pilot determine free carbon emission quotas for the regulated firms based on their historical carbon emission data, the carbon emission level of the industry, and the potential of future carbon emission reduction of the industry. Second, if the actual carbon emission of the firms is greater than the free carbon emission quota, the firms need to purchase extra emission rights from the carbon market. When the carbon market’s compliance deadline arrives, firms will more actively participate in carbon emission quota transactions, while firms with surplus carbon emission quotas will sell their carbon emission rights.

### 2.2. Theoretical Hypothesis

Market-based environmental regulatory policies need to rely on well-functioning market mechanisms to play their regulatory role (Kathuria, 2006) [26]. As a market-based environmental regulation policy, CCETP has attracted extensive participation of the regulated firms. Through carbon market tools, which are the mechanism internalizing the external costs of corporate environmental pollution, carbon dioxide emissions are controlled and the goals of environmental protection are achieved. In the process of firms participating in the carbon market, the policy constraining carbon emissions will prompt managers of firms to change production and operating decisions. For firms that focus on the maximization of profit, they have the motivation to increase their investment in technological innovation to improve their production processes and reduce cost pressures brought about by environmental policies (Calel and Dechezlepretre, 2016; Ambec et al., 2013; Lanoie et al., 2008) [2,8,27].

**Figure 1:**
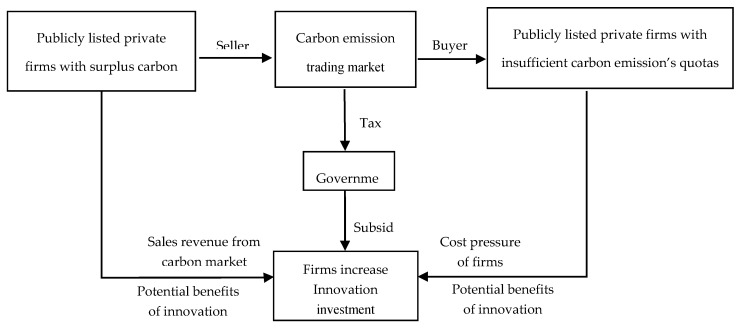
The influence mechanism through which CCETP affects the innovation input of publicly listed private firms.

In the carbon market, the supply and demand sides of the carbon quota will voluntarily adjust the R&D investment of firms under the influence of the market mechanism and, ultimately, affect firm innovation. First, let us analyze the influence mechanism of the carbon market from the supply side. Under the influence of the cap and trading system, publicly listed private firms with more carbon emission quotas can obtain additional income from the carbon market to enhance their innovation ability. Although the high uncertainty and high cost in the early stage of technological innovation often suppress firm innovation, the promotion of innovation brought by the increase in R&D investment can help to reduce carbon emissions under the same output, improve the supply capacity for carbon emission rights, and gain the potential benefits of innovation (Fan et al., 2016) [28]. In addition, the government can obtain additional tax revenue by advancing carbon emission transactions and then use part of these taxes to directly reward the regulated firms or provide subsidies for energy conservation and emission reduction technology. It will further stimulate firm innovation, improve production efficiency, and increase the added value of products (Demirel and Kesidou, 2011) [29]. Second, we analyze the influence mechanism of the carbon market from the demand side. Under the influence of CCETP, if the actual carbon emission of the regulated firms exceeds the free carbon emission quota, they may choose two types of strategies: (1) If regulated firms maintain or expand the original production scale, they have to purchase extra carbon emission quotas from the carbon market to meet the policy requirements of the supervising authorities. However, this will increase the cost of the firms and reduce their market competitiveness. (2) If regulated firms choose to reduce their output and control the total carbon emission within the free carbon emission quota allocated, it will reduce their income, profit, and future market competitiveness. However, no matter which one the firms choose, they will face the negative impact of environmental regulation when the production technologies and innovation capabilities remain unchanged. Only by actively increasing R&D investment and promoting technological innovation can firms radically reduce their carbon emission. The two theoretical mechanisms above are shown in Figure 1.

Based on two theoretical mechanisms, it is expected that CCETP will have a positive effect on innovation input. To verify whether this impact exists, we propose hypothesis H1a:

**H1a:**  CCETP has a positive impact on the innovation input of publicly listed private firms.

It is also pointed out that CCETP, as a typical market-based environment regulation, can also have a negative impact on the innovation input of publicly listed private firms because environmental regulation has crowding-out effects on firm investment (Kneller and Manderson, 2012) [3]. Restrictions on the total amount of carbon emissions may reduce the economic activities and operating performance of the publicly listed private firms, thereby affecting the firms’ R&D input. Therefore, hypothesis H1b is as follows:

**H1b:**  CCETP has a negative impact on the innovation input of publicly listed private firms.

Publicly listed private firms respond relatively more sensitively to external policy uncertainties under China’s public ownership system. At the same time, the political background of publicly listed private firm executives can also enhance the acquired relationship between publicly listed private firms and the government to a certain extent. It may affect the effect of external regulation on firm behavior. Specifically, firstly, the enhancement of political connection can promote the R&D investment of firms by enhancing the ability of publicly listed private firms to obtain government subsidies (Faccio and Masulis, 2006) [23] and tax incentives and break through financing constraints (Li et al., 2008; Gao et al., 2019) [22,30]. Moreover, the final carbon emission reduction effect will directly affect the assessment and promotion of local officials. Therefore, political connection will transfer the pressure of officials to firms and make them more involved in carbon emission reduction. In order to reduce the negative impact of carbon emissions on firms, publicly listed private firms will increase R&D investment. Secondly, the improvement of political connection may enable the firms to obtain a relatively favorable institutional environment, increase their dependence on government resources and policies (Chen and Wu, 2011) [31], reduce their reliance on firm innovation, and inhibit the increase in R&D investment. In addition, in order to maintain the political connection between the firms and the government, the firms may take additional social responsibilities such as charity and increasing employment, which will have a crowding-out effect on the expansion of R&D investment (Kneller and Manderson, 2012) [3]. It could weaken the positive effect of CCETP on the innovation input of the regulated firms. Therefore, under the influence of the two types of influence mechanisms, the final moderating effect of political connection needs to be further tested. We propose H2a and H2b:

**H2a:**  Political connection will enhance the promotion effect of CCETP on firm innovation input when other conditions remain unchanged.

**H2b:**  Political connection will inhibit the promotion effect of CCETP on firm innovation input when other conditions remain unchanged.

## 3. Research Design and Main Model

### 3.1. Selection of Samples

In China’s economic system, the public sector of the economy dominates the whole economy to some degree. Therefore, firms are often classified according to the nature of their ownership. Chinese firms can generally be divided into state-owned firms, nonstate-owned firms, and foreign-funded firms, while nonstate-owned firms can all be called private firms. The private firms in this paper are also classified according to the nature of firm ownership. In China’s A-share market, publicly listed firms can be divided into publicly listed state-owned firms and publicly listed private firms. This paper only studies the effect of CCETP on publicly listed private firms. This paper takes publicly listed private firms listed on the Shanghai and Shenzhen A-share markets in China from 2010 to 2014 as research samples. Firstly, there are three reasons for setting the window period to 2010–2014. (1) The window period selected can identify policy effects better and exclude the noise interference that may exist for a long period. (2) The data of R&D investment before 2010 are missing too much information. (3) The window period can evaluate the direct policy effect of CCETP on firms’ innovation input. Secondly, we deal with data in the following steps, excluding (1) listed firms after 2010, (2) sample firms of ST on the A-share market. (3) firms of the financial industry, and (4) firms with missing data. Then, 2974 observations were obtained. Then, we selected the regulated firms that were first included in the quota management list in the 7 pilots as the treatment group, and deleted the samples that were removed from the list in the following years, as well as the samples whose registration place and actual management place were not the same. Finally, due to the lack of firm R&D input data, we ended up with 23 regulated publicly listed private firms, with 105 effective observations that were nonbalanced panel data. In order to eliminate the influence of outliers, this paper makes a tailing treatment for all variables at 1% and 99% quantiles.

The data sources of this paper are as follows. Firm R&D investment intensity, executive political background, and other firm-level characteristic variable data, as well as data of the provincial level are from the CSMAR database. The CSMAR(China Stock Market&Accounting Research Database) database is a research accurate database in the economic and financial field which is developed based on China’s actual national conditions. Regulated firms were sorted out of the website of the Development and Reform Commissions of the seven pilots.

### 3.2. Variables Explanation

In this paper, the impact of CCETP on innovation input will be checked. The main variables are as follows.

The dependent variable is the intensity of R&D investment (RD). This paper mainly uses the intensity of R&D investment as a proxy variable for the innovation input of China’s publicly listed private firms.

The independent variable is the interactive item TREAT × POST, whose coefficient reflects the change in R&D investment between regulated and nonregulated firms before and after CCETP, where TREAT indicates whether the firm is a regulated firm included in the carbon emission quota management list, and POST indicates before or after the establishment year of CCETP. Since the establishment of CCETP was mainly in 2013, this paper takes 2013 as the year in which policy shocks occurred; the years before 2013 are the prepilot period, the years after 2013 are the postpilot period.

Existing literature has studied the influencing factors of firm innovation input (Gao et al., 2019; Chang et al., 2015; Ferris et al., 2016) [30,32,33]. According to them, we determined the control variables and moderating variables in this paper. The main explanations of them are in Table 1.

### 3.3. Empirical Model

In order to test H1, we construct the following Model (1). In Model (1), we used the difference-in-differences (DID) method, which is mainly aimed at verifying whether CCETP can promote publicly listed private firms to increase innovation input or not. In order to distinguish between the treatment group and the control group, and to identify whether the R&D investment of different firms is affected by the policy intervention before and after policy implementation, two dummy variables were needed to divide the samples into different groups. Therefore, the DID model in this paper needs relatively more dummy variables.

(1) $$RD_{it}=\gamma TREAT_{i}+\beta POST_{t}+\delta TREAT_{i}\times POST_{t}+X_{it}^{\prime }\theta +u_{s}+v_{t}+\sigma_{k}+\varepsilon_{itsk}$$

where $RD_{it}$ is the dependent variable, which measures the firm’s investment in innovation; $TREAT_{i}\times POST_{t}$ is the independent variable, whose coefficient is the policy effect that this paper focuses on; $TREAT_{i}$ is a dummy variable that denotes whether the firm is included in the carbon emission pilot list, $TREAT_{i}$ is 1 if the firm *i* is a regulated firm, otherwise 0. The variable $POST_{t}$ indicates whether the time is after 2013 or not; the value is 0 if before 2013, otherwise 1. $X_{it}^{\prime }$ is a control variable changing over time at the firm level. $u_{s}$ is the industry fixed effect, which is used to control industry-level unobservable factors that affect firms’ R&D investment but do not change over time. $v_{t}$ is the year’s fixed effect, which is used to control the impact of time-varying factors at the macro level. $\sigma_{k}$ is the firms’ fixed effect, which is used to control firm-level unobservable factors that affect the firms’ R&D investment but do not change over time. $\varepsilon_{itsk}$ is an error term. To reduce the possible impact of heteroscedasticity and intragroup correlation, this paper uses standard robust errors at the province level.

When existing research constructs political connection indicators, most of the dummy variables are constructed according to whether the chairman or general manager of the firm has a political background. For example, whether the chairman or general manager of the firm works in a government agency or whether he or she is an industry supervisor or representative of the people’s congress (Fan et al., 2007; Gao et al., 2019; Ferris et al., 2016) [17,30,33]. Besides, indicators of political connection are generally one-dimensional. Information about personal and organizational relations and personal relations related to privacy is difficult to obtain publicly. Therefore, this paper draws on mainstream practices to construct the dummy variable. In order to test whether the political connection has a moderating effect on the impact of CCETP on publicly listed private firms’ innovation input, we set up Model (2):

(2) $$RD_{it}=\delta_{1}TREAT_{i}\times POST_{t}\times PC_{it}+\delta_{2}TREAT_{i}\times POST_{t}+\delta_{3}TREAT_{i}\times PC_{it}\;+\delta_{4}POST_{t}\times PC_{it}+\alpha PC_{it}+\beta TREAT_{i}+\gamma POST_{t}+X_{it}^{\prime }\theta +u_{s}+v_{t}\;+\sigma_{k}+\varepsilon_{itsk}$$

where $PC_{it}$ is a dummy variable that measures the difference between political connection; if the chairman or general manager of the firm has a political background, the value is 1, otherwise 0.

## 4. Main Results

### 4.1. Summary Statistics

**Figure 2:**
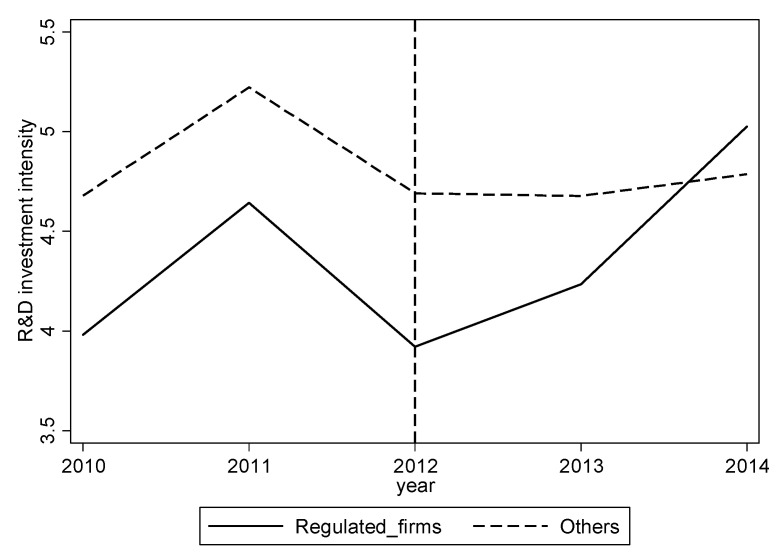
Change of the R&D investment intensity of two types of publicly listed private firms. Source: The original data are obtained from the CSMAR database and the websites of the Development and Reform Commissions of the seven pilots in China and calculated by Stata 14.

Figure 2 shows the trend of R&D investment intensity of regulated and nonregulated publicly listed private firms before and after the establishment of seven pilots in 2013. It shows that before 2012, the intensity of R&D investment of the regulated firms is generally less than that of nonregulated publicly listed private firms, and the two maintain a parallel change trend. However, since 2013, the intensity of the R&D investment of nonregulated firms has not changed significantly. In contrast, for regulated firms, it has increased significantly; especially in 2014, it increased sharply, even surpassed that of nonregulated firms. The different trend of intensity of R&D investment of firms shows that CCETP has a positive effect on the intensity of R&D investment of publicly listed private firms.

Table 2 shows the descriptive statistics of all variables. The results are as follows. First, the minimum value of RD is 0.01 and the maximum value is 25.3, indicating that the R&D investment intensity of publicly listed private firms varies greatly, and the average value (4.58) is greater than the median (3.51), indicating that more than half of the firms’ R&D investment intensity is less than the average. Second, the mean value of the political correlation variable (PC) is 0.602, that is, the chairman or general manager of 60.2% of the firms in the sample has a political background, which shows that publicly listed private firms pay attention to the political connection of executives when selecting their chairman or general manager. Third, the average value of the variable TREAT is 0.035, which indicates that the proportion of the treatment group samples in the whole sample is at a low level (3.5%). In order to avoid the possible deviation from this problem, this paper adopts the PSM–DID method to test the robustness of the effect of CCETP on innovation input of publicly listed private firms.

### 4.2. Main Results

Table 3 reports the results of the impact of CCETP on the R&D investment of publicly listed private firms using the DID method. Column A reports the result without controlling the fixed effects but with all the control variables. It shows that the coefficient of TREAT × POST is 0.699, and it is significant at the level of 5%, indicating that CCETP has a positive effect on the R&D investment of regulated firms. Column B and Column C report the results of controlling for the time fixed effect and the industry fixed effect, respectively. It shows that the coefficient of TREAT × POST is significantly positive. Column D reports the result, controlling for both the time fixed effect and the industry fixed effect. The result shows that CCETP still has a positive impact on the improvement of R&D investment of publicly listed private firms. Column E reports the result, controlling the year fixed effect, industry fixed effect, and firm fixed effect; it is positive and robust with *R*^2^ (0.880). It shows that CCETP, as a type of market-incentive environmental regulation, has a positive effect on the innovation input of publicly listed private firms, which supports H1a.

### 4.3. Robustness Test

#### 4.3.1. Parallel Trend Test

Before the policy shock, the outcome variables of the treatment group and the control group should have parallel trends, which is an important prerequisite for the DID method. In order to test whether the changes in R&D investment of regulated firms and nonregulated firms meet the parallel trend assumption before CCETP was established, this paper adopts Model (3) to analyze the leading and lagging effects of the policy based on the methods of Autor (2003) [34] and He et al. (2016) [35].

(3) $$RD_{it}=\sum_{j=-n}^{m}\lambda_{j}D_{it}\left(t=k_{i}-j\right)+X_{it}^{\prime }\theta +\mu_{s}+v_{t}+\sigma_{k}+\varepsilon_{itsk}$$

In Equation (3), $k_{i}$ represents the time the shock occurs (in 2013). If the year *t* equals to $k_{i}-j$, firm i is a regulated firm; the treatment variable $D_{it}\left(t=k_{i}-j\right)$ equals to 1, otherwise 0. This paper sets the base year as 2010 and, further, leads the treatment variable by 1 year and lags by 1 year, that is, m = 1 and n = 1. $\lambda_{j}$ is the coefficient of treatment variables and, according to the parallel trend, for *j* > 0, $\lambda_{j}$ equals to 0.

In Table 4, it shows that the coefficient of treatment variable Before1 is not significant, which indicates that the R&D investment of this group satisfies the parallel trend before CCETP. Moreover, the coefficients of Current and After1 are significantly positive, indicating that compared with nonregulated publicly listed private firms, the R&D investment of regulated publicly listed private firms has increased significantly after the implementation of the policy. The model satisfies the hypothesis of the parallel trend.

#### 4.3.2. PSM–DID Result

This paper processes the samples with the propensity score matching (PSM) method before performing DID analysis in order to reduce the sample selection bias. Among the seven pilots, publicly listed private firms whose carbon dioxide emissions or energy consumption have reached a certain value have been included in the carbon emission trading quota management list. It is difficult to match firms as the control group within each pilot. Therefore, we match the regulated firms of the pilots with firms of nonpilot provinces. The selection of appropriate matching variables has a critical impact on the quality of the matching between the treatment group and the control group. This paper selects the size of the firm (SIZE), profitability (ROA), time to market (AGE), regional economic development level (GDP), and environmental law enforcement (ELE) as control variables to match the treatment group with the control group among all samples before the establishment of the carbon emission trading pilots (2012). The parallel trend test results show that the absolute value of standardized bias of each feature variable after matching is less than 5%, and the mean t-test result shows that there is no systematic difference between the matched treatment group and the control group (see Appendix A, Table A1). Moreover, the sample satisfies the conditional independent distribution hypothesis. Using the matched sample to regress the baseline model, it is found that CCETP still has a significantly positive impact on the innovation input of publicly listed private firms (0.374). The results are reported in Column A of Table 5.

#### 4.3.3. Change the Measurement of Dependent Variable

In this part, we take the natural logarithm of total R&D investment as the dependent variable to conduct a robustness test. The results are shown in Column B of Table 5. It shows that the coefficient of TREAT × POST is still positive, and the result is robust.

#### 4.3.4. Shorten the Sample Period

Shortening the sample period before and after policy implementation will help to better identify the actual effect of the policy. This paper selects the sample from 2012 to 2013 to reexamine the effect of CCETP on the innovation input of publicly listed private firms. The result is reported in Column C of Table 5. It shows that the coefficient of TREAT × POST is still robust.

#### 4.3.5. Remove Samples of Chongqing and Tianjin

Among the seven carbon pilots, the carbon emission and trading volumes in Chongqing and Tianjin are close to 0. To avoid bias, we eliminated samples of these two pilots to make a robustness test. The result is reported in Column D of Table 5. The result is still robust.

#### 4.3.6. Extend the Sample Period to 2010–2017

The improvement of the publicly listed private firms’ ability to control carbon emissions after 2015 has led to a decrease in carbon emission transactions. It may make the positive effect of CCETP on innovation input gradually stabilize and further affect the evaluation of policy effects. The baseline regression in this paper only selects samples from 2010 to 2014; we extended the sample time to 2010–2017 (the carbon trading and pilot system ended in 2017) to test for robustness. The result is reported in Column E of Table 5, and it is still a positive effect.

## 5. Further Study

### 5.1. Comparison of the Impact of CCETP on Innovation Input and Innovation Output

Existing literature usually measures innovation of firms in terms of input and output. Innovation input is mainly measured by the amount of R&D input and intensity of R&D input. Innovation output mainly includes the number of patent applications or authorizations and the output of new products (Brunnermeier and Cohen, 2003) [36]. Due to the low R&D efficiency of Chinese firms and the high uncertainty of their innovation output (Ouyang et al., 2020) [5], the intensity of R&D investment can directly measure the firms’ response to market-based environmental regulation. Therefore, we mainly used the intensity of R&D investment as a proxy variable for firm innovation input in the baseline regression. Next, we took the number of invention patent applications of the regulated firms as a proxy variable for firm innovation output and examined the impact of CCETP on firm innovation output. The results are shown in Appendix A, Table A2. Columns A–C report the impact of CCETP on firm patents in the current period, and Columns D–F report the impact of CCETP on patents that are lagged by one period. It shows that TREAT × POST has no significant impact on firm innovation output, which is consistent with theoretical expectations, and indicates that R&D efficiency in China is still relatively low. Therefore, we can make the conclusion that CCETP has led to an increase in firm innovation input, while it does not bring about a synchronous increase in firms’ innovation output.

### 5.2. Moderating Effect of Political Connection on the Impact of CCETP

Column A of Table 6 reports the result of the moderating effect of political connection (the coefficient of TREAT × POST × PC). The result shows that the coefficient is significantly positive (0.724), which supports H2a. It indicates that when CCETP increases the innovation input of the regulated publicly listed private firms, the political connection of their executives is conducive to enhancing the positive effect of CCETP on the R&D investment of the regulated publicly listed private firms. That is to say, CCETP will have a greater significant effect on the innovation input of regulated publicly listed private firms that have political connection.

Compared with nonregulated firms and firms without political connection, CCETP has a significant positive impact on the innovation of the regulated publicly listed private firms and the regulated firms with political connection. To obtain the potential benefits of policy preference and with the incentives of subsidies for energy conservation and emission reduction, firms with political connection are more willing to actively participate in the carbon market and improve innovation. Therefore, compared with publicly listed private firms without political connection, CCETP has a stronger promotion effect on innovation on publicly listed private firms with political connection.

### 5.3. Heterogeneity of the Moderating Effect of Political Connection

From the above results, it is found that there is a significant moderating effect of political connection on the impact of CCETP on firm innovation input. It is believed that there may be two factors that affect the moderating effect. Therefore, we conducted a further heterogeneity test. First, we divided the samples into a high environmental protection input group and a low environmental protection input group based on the average value of the proportion of the energy-saving and environmental protection expenditure of each pilot. The results are reported in Columns B andC of Table 6. It shows that in the high environmental protection input group, the coefficient of TREAT × POST × PC is significantly positive at the level of 1% (0.892), and has a greater effect than the one of the full sample (0.621). In contrast, in the low environmental protection input group, the coefficient of TREAT × POST × PC is significantly negative. The reason may be that in regions with high fiscal investment in environmental protection, publicly listed private firms with political connection have stronger enforcement of policies. Therefore, they can obtain more policy support and increase R&D investment to create technological innovation. Conversely, the firms’ investment in innovation is low or even has a tendency to decrease, showing a negative moderating effect (Agrawal and Knoeber, 2001; Claessens et al., 2008; Li et al., 2008) [20,21,22]. At present, China is in a period of transformation and upgrading. There are significant differences in the level of economic development, innovation infrastructure, and policy environment in different regions (Gao et al., 2017) [37]. The proportion of regional energy conservation and environmental protection expenditure of local governments, to some extent, can reflect the local government’s efforts to control pollution. Among these expenditures are special financial expenditures to support energy conservation, emission reduction, and cleaner production that can attract firms to obey the requirements of government policies to protect the environment. Second, we divided the sample into large-scale publicly listed private firms and small-scale publicly listed private firms, according to the median of the scale of publicly listed private firms. The results are reported in Columns D and E of Table 6. The results show that in the large publicly listed private firm group, the coefficient of TREAT × POST × PC is significantly positive, while in the small publicly listed private firm group, it is significantly negative. This may be because those large publicly listed private firms that have stronger R&D capabilities and more willingness to invest are more closely connected with the government. Therefore, when faced with carbon market constraints, they are more inclined to increase their R&D investment by convenient financing through political connection and reduce the negative impact of environmental regulation through technological innovation. In contrast, for small firms with weak innovation capabilities and low antirisk ability, they are more inclined to make up for firm losses and maintain normal operations through the profit of political connection.

## 6. Conclusions and Policy Implications

Using the sample of regulated publicly listed private firms in China from 2010 to 2014, we took China’s carbon emissions and trading pilot (CCETP) as a quasi-natural experiment to examine the policy impact of CCETP on the innovation input of publicly listed private firms and the moderating effect of the firms’ political connection on the relationship between them, based on a difference-in-differences model. Conclusions and implications are as follows.

First, CCETP has effectively promoted the innovation input of Chinese publicly listed private firms. The results of this paper fully verify that market-based environmental regulation can significantly induce publicly listed private firms to increase R&D investment and achieve a win–win between environmental protection and innovative development. The weak Porter hypothesis has, again, been verified in this paper. In order to reduce the negative impact of CCETP on publicly listed private firms’ R&D investment, the Chinese government should strengthen cooperation with publicly listed private firms in the construction of China’s carbon market and provide more convenience and policy guidance to them to give full play to the role of the national carbon market. Moreover, this article verifies that CCETP has promoted the win–win development of Chinese publicly listed private firms and the environment. Therefore, the Chinese government should continue to intensify the construction of the carbon market, improve the quota and trading system, and improve guidance policies. Only by these strategies can the carbon market have a better role in stimulating the innovation of firms and reducing carbon emissions.

Second, there are differences in the impact of CCETP on the innovation input and output of publicly listed private firms. It shows that CCETP can effectively increase the innovation input of publicly listed private firms; however, the impact on the output of firms’ innovation is not obvious due to the low efficiency of R&D output in China. The government needs to build more public service platforms to improve the efficiency and transformation of R&D and formulate effective strategies to accelerate the actual conversion of R&D investment to innovation output. This article further confirms the low R&D efficiency of Chinese publicly listed private firms at present, which is consistent with existing research (Liu et al., 2020; Zhang et al., 2019) [13,15], indicating that it is a long process for Chinese firms to shift from attaching importance to increasing R&D investment to innovation output. How to transform human, capital, and resource input into innovation output is an urgent problem for firms to solve. At present, China is experiencing an economic transformation, so it requires more technological innovation to promote the transformation. During this process, the government needs to provide public services, as well as to promote the technological innovation of the firms.

Third, the political connection of a firm’s executives has a positive moderating effect on the impact of CCETP on innovation input. The carbon market has a stronger innovation promotion effect on publicly listed private firms with stronger political connection. At the same time, the political connection is more likely to generate positive incentives for the carbon market to promote firm innovation input in regions with high environmental protection investment and large firms. The government should pay more attention to the institutional design and business environment of the carbon market to reduce the burden on firms with less political connection. Moreover, convenient financing and innovation subsidies should be provided to small- and medium-sized publicly listed private firms to stimulate the innovation vitality of the firms. For publicly listed private firms, they can take advantage of their political connection, without breaking the law, to enhance their competitiveness in the market.

In this paper, there are still some limitations in our study that can be improved in further discussion. First, in our paper, we mainly focused on the impact of CCETP on the innovation input of all publicly listed private firms in the seven carbon pilots. In fact, we did not consider the specific circumstances of each carbon pilot to some extent because of limited data on the regulated publicly listed private firms by CCETP. In the robustness test, we only excluded the Chongqing and Tianjin pilots and did not further differentiate and quantify the activity of the remaining five pilots. Therefore, we cannot distinguish the impact of CCETP on firm innovation input in each pilot, which can be improved in the future. Second, this paper has deficiencies in the construction of key variable indicators. Specifically, we did not use the dimensions of political connection but a dummy variable. This can be carried out from other perspectives in future research.

## Figures and Tables

**Table 1:** Definition and description of main variables.

Types	Variable	Description
Dependent variable	RD	R&D investment intensity: R&D spending/Operating income (%)
Independent variable	TREAT	The value is 1 if the firm is in the CCETP list, otherwise 0.
	POST	If before 2013, the value is 0, otherwise 1
Control variable	SIZE	Firm size: Total assets of firm (in log)
	LEV	Solvency: Total liability/Total assets
	ROA	Profitability: Net profit after tax/Total assets
	FIXEDPP	Capital density: Net fixed assets per capita (in log)
	MB_ratio	Book-to-market ratio
	COCEN	Equity concentration: Shareholding ratio of the largest shareholder (%)
	MSH	Number of management shares/Total shares
	INDEP	Number of independent directors/Number of directors
	DUAL	Whether the chairman and general manager serve concurrently; if so, the value is 1, otherwise 0.
	AGE	Firm listing years (in log)
Moderating variable	PC	Executive political connection: Whether the firm’s chairman or general manager had a political background; the value is 1 if executives have served in government agencies at all levels, former or current National People’s Congress’s deputies, members of the Chinese People’s Political Consultative Conference, otherwise 0.

Notes: RD: R&D investment intensity; TREAT: the dummy variable whether the firm is in the CCETP list; POST: the dummy variable whether time is before 2013; SIZE: firm size; LEV: asset-liability ratio; ROA: return on total assets; FIXEDPP: capital density; MB_ratio: book-to-market ratio; COCEN: ownership concentration; MSH: the proportion of management shares; INDEP: the proportion of independent directors; DUAL: the dummy variable whether chairman and general manager serve concurrently; AGE: length of firm establishment; PC: the dummy variable whether the firm has executive political connection.

**Table 2:** Descriptive statistics.

Variables	Observations	Mean	Standard Deviation	Minimum	Median	Maximum
RD	2974	4.580	4.382	0.010	3.510	25.300
TREAT	2974	0.035	0.185	0	0	1
POST	2974	0.473	0.499	0	0	1
SIZE	2974	7.689	0.930	6.142	7.538	11.733
LEV	2974	0.341	0.198	0.045	0.317	0.877
ROA	2974	0.056	0.053	−0.145	0.053	0.213
FIXEDPP	2974	12.229	0.924	9.650	12.287	15.218
MB_ratio	2974	0.671	0.539	0.106	0.541	4.838
COCEN	2974	33.368	14.225	8.448	31.769	74.095
MSH	2974	0.228	0.231	0	0.144	0.714
INDEP	2974	0.372	0.052	0.333	0.333	0.571
DUAL	2974	0.377	0.485	0	0	1
AGE	2974	1.631	0.780	0	1.609	3.045
PC	2974	0.602	0.490	0	1	1

Notes: RD: R&D investment intensity; TREAT: the dummy variable whether the firm is in the CCETP list; POST: the dummy variable whether time is before 2013; SIZE: firm size; LEV: asset-liability ratio; ROA: return on total asset; FIXEDPP: capital density; MB_ratio: book-to-market ratio; COCEN: ownership concentration; MSH: the proportion of management shares; INDEP: the proportion of independent directors; DUAL: the dummy variable whether chairman and general manager serve concurrently; AGE: length of firm establishment; PC: the dummy variable whether the firm has executive political connection. The original data are obtained from the CSMAR database and the websites of the Development and Reform Commissions of the seven pilots in China and calculated by Stata 14.

**Table 3:** The results of the impact of CCETP on the R&D investment of publicly listed private firms.

Variable	A	B	C	D	E
	RD	RD	RD	RD	RD
TREAT × POST	0.699 **	0.641 **	0.711 ***	0.656 ***	0.621 ***
	(0.295)	(0.252)	(0.153)	(0.128)	(0.100)
TREAT	0.128	0.198	−0.228	−0.196	0.000
	(0.279)	(0.269)	(0.285)	(0.267)	(.)
POST	0.541 ***	0.000	0.357 ***	0.000	0.000
	(0.121)	(.)	(0.111)	(.)	(.)
SIZE	0.383 **	0.365 **	0.367 **	0.361 *	−0.343
	(0.150)	(0.151)	(0.180)	(0.188)	(0.264)
LEV	−6.652 ***	−6.087 ***	−5.594 ***	−5.097 ***	−2.246 **
	(1.277)	(1.197)	(1.066)	(1.009)	(0.900)
ROA	−9.595 ***	−8.155 ***	−9.307 ***	−8.172 ***	−9.473 ***
	(1.851)	(1.780)	(2.212)	(2.091)	(2.149)
FIXEDPP	−1.015 ***	−1.030 ***	−0.268 *	−0.281 *	−0.111
	(0.175)	(0.170)	(0.151)	(0.150)	(0.101)
MB_ratio	−0.806 **	−0.964 **	−0.579 **	−0.747 **	−0.002
	(0.316)	(0.357)	(0.281)	(0.331)	(0.238)
COCEN	−0.045 ***	−0.048 ***	−0.019 **	−0.022 ***	−0.019 **
	(0.013)	(0.013)	(0.007)	(0.008)	(0.008)
MSH	1.122	0.795	0.550	0.269	1.911
	(0.751)	(0.755)	(0.498)	(0.542)	(1.347)
INDEP	2.288	2.303	2.033	2.008	−0.444
	(1.396)	(1.424)	(1.410)	(1.406)	(0.835)
DUAL	0.376*	0.359	0.185	0.178	0.095
	(0.214)	(0.220)	(0.163)	(0.166)	(0.193)
AGE	−0.022	−0.441	0.032	−0.331	0.165
	(0.269)	(0.320)	(0.242)	(0.324)	(0.445)
Constant	17.430 ***	17.266 ***	7.135 ***	6.983 ***	5.837 **
	(2.576)	(2.501)	(1.643)	(1.655)	(2.764)
Year fixed effect	No	Yes	No	Yes	Yes
Industry fixed effect	No	No	Yes	Yes	Yes
Firm fixed effect	No	No	No	No	Yes
observations	2974	2974	2974	2974	2974
Adj *R*^2^	0.187	0.195	0.431	0.436	0.880

Notes: RD: R&D investment intensity; TREAT: the dummy variable whether the firm is in the CCETP list; POST: the dummy variable whether time is before 2013; SIZE: firm size; LEV: asset-liability ratio; ROA: return on total assets; FIXEDPP: capital density; MB_ratio: book-to-market ratio; COCEN: ownership concentration; MSH: the proportion of management shares; INDEP: the proportion of independent directors; DUAL: the dummy variable whether chairman and general manager serve concurrently; AGE: length of firm establishment. The robust standard errors of the province level are reported in parentheses. ***, **, and * represent significance at the levels of 1%, 5%, and 10%, respectively. The original data are obtained from the CSMAR database and the websites of the Development and Reform Commissions of the seven pilots in China and are calculated by Stata 14.

**Table 4:** Parallel trend test.

Variable	RD
Before 1	−0.062
	(0.213)
Current	0.286 *
	(0.166)
After 1	1.169 ***
	(0.296)
Constant	5.767 **
	(2.755)
Control variables	Yes
Year fixed effect	Yes
Industry fixed effect	Yes
Firm fixed effect	Yes
Observations	2974
Adj R^2^	0.880

Notes: RD: R&D investment intensity; Before1: the coefficient when the treatment variable is lead by one period; Current: the coefficient when the treatment variable is current period data; After1: the coefficient when the treatment variable is lagged by one period. The robust standard errors of the province level are reported in parentheses. ***, **, and * represent significance at the levels of 1%, 5%, and 10%, respectively. The original data are obtained from the CSMAR database and the websites of the Development and Reform Commissions of the seven pilots in China and calculated by Stata 14.

**Table 5:** Robustness test.

Variable	A	B	C	D	E
	RD	Ln_RDSpend	RD	RD	RD
TREAT × POST	0.374 ***	0.174 **	0.272 *	0.626 ***	0.393 ***
	(0.106)	(0.075)	(0.138)	(0.089)	(0.118)
Constant	6.150 ***	12.180 ***	7.619	5.447 *	4.349 **
	(1.819)	(1.669)	(8.658)	(2.758)	(1.607)
Control variable	Yes	Yes	Yes	Yes	Yes
Year fixed effect	Yes	Yes	Yes	Yes	Yes
Industry fixed effect	Yes	Yes	Yes	Yes	Yes
Firm fixed effect	Yes	Yes	Yes	Yes	Yes
Observations	1767	2974	1429	2917	5052
Adj R^2^	0.863	0.871	0.942	0.882	0.834

Notes: RD: R&D investment intensity; TREAT: the dummy variable whether the firm is in the CCETP list; POST: the dummy variable whether time is before 2013; Ln_RDSpend: total R&D investment. The robust standard errors of the province level are reported in parentheses. ***, **, and * represent significance at the levels of 1%, 5%, and 10%, respectively. The original data are obtained from the CSMAR database and the websites of the Development and Reform Commissions of the seven pilots in China and calculated by Stata 14.

**Table 6:** Moderating effect of political connection.

Variables	Full Sample	Firms with High Environmental Protection Investment	Firms with Low Environmental Protection Investment	Large Firms	Small Firms
	A	B	C	D	E
	RD	RD	RD	RD	RD
TREAT × POST × PC	0.724 **	0.892 ***	−1.073 **	0.751 *	−0.949 *
	(0.347)	(0.278)	(0.503)	(0.408)	(0.553)
TREAT × POST	0.234	0.295 *	1.158 **	0.186	1.224 ***
	(0.179)	(0.147)	(0.414)	(0.318)	(0.402)
POST × PC	0.163	0.289	−0.121	−0.071	0.156
	(0.193)	(0.182)	(0.416)	(0.232)	(0.323)
TREAT × PC	−0.074	−0.544	0.000	0.649	0.000
	(0.405)	(0.804)	(.)	(0.559)	(.)
PC	−0.133	0.355	−0.645	−0.324	−0.442
	(0.320)	(0.400)	(0.457)	(0.322)	(0.546)
Constant	6.190 **	11.307 ***	6.723 ***	12.528 ***	5.842
	(2.756)	(3.733)	(2.003)	(3.876)	(6.082)
Control variables	Yes	Yes	Yes	Yes	Yes
Year fixed effect	Yes	Yes	Yes	Yes	Yes
Industry fixed effect	Yes	Yes	Yes	Yes	Yes
Firm fixed effect	Yes	Yes	Yes	Yes	Yes
observations	2974	1640	1334	1473	1501
Adj R^2^	0.880	0.878	0.889	0.888	0.891

Notes: RD: R&D investment intensity; TREAT: the dummy variable whether the firm is in the CCETP list; POST: the dummy variable whether time is before 2013; PC: the dummy variable whether the firm has executive political connection. The robust standard errors of the province level are reported in parentheses. ***, **, and * represent significance at the levels of 1%, 5%, and 10%, respectively. The original data are obtained from the CSMAR database and the websites of the Development and Reform Commissions of the seven pilots in China and calculated by Stata 14.

## Appendix A

**Table A1:** Balance test.

Variables	Unmatched/Matched	Treatment Group	Control Group	%bias	T Value	*p* Value
SIZE	U	8.162	7.776	41.100	1.940	0.053
	M	8.162	8.177	−1.600	−0.050	0.960
ROA	U	0.049	0.047	3.000	0.150	0.880
	M	0.049	0.046	4.200	0.140	0.891
AGE	U	1.797	1.798	−0.200	−0.010	0.994
	M	1.797	1.817	−3.200	−0.110	0.914
GDP	U	10.710	10.254	80.600	3.390	0.001
	M	10.710	10.690	3.500	0.160	0.875
ELE	U	0.043	0.213	−52.200	−1.970	0.049
	M	0.043	0.054	−3.300	−0.170	0.868

Notes: SIZE: firm size; ROA: return on total assets; AGE: length of firm establishment; GDP: regional Gross National Product; ELE: strength of environmental enforcement. The original data are obtained from the CSMAR database and the websites of the Development and Reform Commissions of the seven pilots in China and calculated by Stata 14.

**Table A2:** Impact of China’s carbon emissions and trading pilot (CCETP) on innovation output.

Variable	A	B	C	D	E	F
	Ln_Apply	Ln_ApplyGrant	Ln_IApply	Ln_Apply	Ln_ApplyGrant	Ln_IApply
TREAT × POST	0.249	0.075	0.292	0.201 **	0.123	0.086
	(0.165)	(0.193)	(0.264)	(0.074)	(0.080)	(0.158)
SIZE	0.300 *	0.271 *	0.228 **	0.297	0.301	0.195
	(0.150)	(0.156)	(0.103)	(0.183)	(0.185)	(0.155)
LEV	−0.300	−0.234	−0.317	−0.535	−0.516	−0.459
	(0.287)	(0.274)	(0.240)	(0.611)	(0.548)	(0.583)
ROA	0.647	0.547	0.611	−0.980	−0.985	−0.427
	(0.866)	(0.859)	(0.567)	(0.777)	(0.969)	(0.743)
FIXEDPP	−0.118 *	−0.127 **	−0.052	0.017	−0.026	0.008
	(0.063)	(0.057)	(0.049)	(0.053)	(0.052)	(0.057)
MB_ratio	−0.041	−0.022	−0.110	0.095	0.093	0.052
	(0.094)	(0.099)	(0.079)	(0.134)	(0.123)	(0.165)
COCEN	−0.007	−0.008	0.002	−0.003	−0.003	0.003
	(0.008)	(0.008)	(0.004)	(0.006)	(0.006)	(0.004)
MSH	0.211	0.310	0.189	0.009	0.065	−0.147
	(0.364)	(0.365)	(0.266)	(0.260)	(0.304)	(0.278)
INDEP	0.084	0.106	−0.341	−1.370	−1.325	−0.853
	(0.572)	(0.609)	(0.568)	(0.929)	(1.020)	(0.762)
DUAL	−0.040	−0.010	0.001	0.031	0.023	−0.001
	(0.088)	(0.083)	(0.078)	(0.100)	(0.122)	(0.074)
AGE	0.404 **	0.426 ***	0.238	0.717 **	0.731 **	0.466
	(0.148)	(0.135)	(0.142)	(0.343)	(0.310)	(0.341)
Constant	1.191	1.932	−0.048	0.202	1.257	−1.324
	(1.527)	(1.377)	(1.207)	(1.560)	(1.513)	(1.416)
Year fixed effect	Yes	Yes	Yes	Yes	Yes	Yes
Industry fixed effect	Yes	Yes	Yes	Yes	Yes	Yes
Firm fixed effect	Yes	Yes	Yes	Yes	Yes	Yes
observations	2974	2974	2974	2113	2113	2113
Adj R^2^	0.734	0.724	0.741	0.724	0.726	0.721

Notes: RD: R&D investment intensity; TREAT: the dummy variable whether the firm is in the CCETP list; POST: the dummy variable whether time is before 2013; SIZE: firm size; LEV: asset-liability ratio; ROA: return on total assets; FIXEDPP: capital density; MB_ratio: book-to-market ratio; COCEN: ownership concentration; MSH: the proportion of management shares; INDEP: the proportion of independent directors; DUAL: the dummy variable whether chairman and general manager serve concurrently; AGE: length of firm establishment; Ln_Apply: number of patent applications; Ln_ApplyG: number of granted patents; Ln_IApply: number of invention patent applications. The robust standard errors of the province level are reported in parentheses. ***, **, and * represent significance at the levels of 1%, 5%, and 10%, respectively. The original data are obtained from the CSMAR database and the websites of the Development and Reform Commissions of the seven pilots in China and calculated by Stata 14.
